# An Acoustic Localization Sensor Based on MEMS Microphone Array for Partial Discharge

**DOI:** 10.3390/s23031077

**Published:** 2023-01-17

**Authors:** Jiaming Yan, Caihui Chen, Zhipeng Wu, Xiaoxia Ding, Liang Lou

**Affiliations:** 1 School of Microelectronics, Shanghai University, Shanghai 201800, China; 2 The Shanghai Industrial μTechnology Research Institute, Shanghai 201899, China

**Keywords:** partial discharge, acoustic localization sensor, microphone array, beam-forming

## Abstract

Partial discharge (PD) localization is important for monitoring and maintaining high-voltage equipment, which can help to prevent accidents. In this work, an acoustic localization sensor based on microelectromechanical system (MEMS) microphone array is proposed, which can detect and locate the partial discharge through a beam-forming algorithm. The MEMS microphone array consists of eight commercial MEMS microphones (SPV08A0LR5H-1, Knowles Electronics, IL, USA) with an aperture size of about 0.1 m × 0.1 m, allowing for a small hardware size and low cost. In order to optimize the acoustic performance of the array, a random array topology is designed. The simulation analysis indicates that the designed random topology is superior to several commonly used topologies. In terms of the localization algorithm, a deconvolution method called Fourier-based fast iterative shrinkage thresholding algorithm (FFT-FISTA) is applied. Simulation and experiment results demonstrate that FFT-FISTA used in the proposed acoustic localization sensor has significant advantages over the conventional beam-forming algorithm on spatial resolution and sidelobe suppression. Experimental results also show that the average localization error of the proposed scheme is about 0.04 m, which can meet the demands of practical application.

## 1. Introduction

Insulation failure in the power system will lead to equipment damage and shutdown, causing huge economic losses and even safety accidents. Partial discharge (PD) is the main indicator of insulation degradation [1]. Therefore, early attention to information about PD can help avoid insulation failures. Partial discharges cause complex physical and chemical changes [2], such as pulse current [3], ultra-high frequency (UHF) electromagnetic waves [4,5], acoustic wave [6,7,8], optical wave [9], gas [10], etc., which can be used as indicators for PD detection. When the presence of PD is detected, it is necessary to confirm its location. The location information can offer suggestions for defect severity analysis and maintenance decisions. Two effective and well-liked methods for locating PDs are acoustic and UHF methods. The UHF method has high detection sensitivity. UHF sensors are usually placed inside power transformers and gas-insulated switchgear (GIS), providing higher localization accuracy compared to the acoustic method [11,12]. If the detection is performed outside of the equipment, the UHF sensor may be subjected to external interferences, such as communication systems [13]. Another problem is the expensive cost due to the high sampling rate required for UHF systems. In comparison, the acoustic method is widely noticed for its low cost, ease of use, and great robustness to electromagnetic interferences. Acoustic sensors can be easily mounted on the outside of equipment or used as a portable, contactless device to provide security to the user [14]. The technique of PD acoustic localization has achieved many exciting outcomes over the years and is able to meet the needs of the application.

The acoustic localization method utilizes a set of acoustic sensors arranged at various positions to receive acoustic signals and determines the location of the source by processing the acquired data. Some studies are based on time-delay estimation [15,16]. The localization equations can be established according to the time difference of arrival (TDOA) of the acoustic waves to various sensors. The PD location can be obtained by solving these equations. This approach is particularly sensitive to TDOA errors, and it is essential to reliably estimate TDOA in complicated situations (low signal-to-noise ratio; absorption, reflection, refraction, and attenuation in the transmission of acoustic waves, etc.). Several researchers focus on advanced array signal processing techniques. Luo et al. measure the direction of arrival (DOA) of the PD acoustic wave by using an ultrasonic array, and an additional UHF system is used to measure the distance between the PD source and the sensor and obtain 3D coordinates for the PD [17,18]. Another method is to use multiple array platforms to measure the DOAs of the acoustic waves separately and solve for the coordinates in combination with the geometric principle [19,20]. Finding the 3D coordinates of PDs seems to be a complex task. In recent years, the acoustic visualization technique is used in the detection and localization of PDs, especially in air-insulated substations. This technique establishes a mapping relationship between the information of planar source distribution measured by an sensor array and the actual image captured by an optical camera [21]. It can provide a more intuitive and convenient way for the diagnosis of insulation status, which has a wide application prospect [22]. Dong et al. propose an ultrasonic sensor array with 31 sensors arranged in a double-helix arrangement, and use a high-resolution spatial spectrum estimation method combined with an image fusion technique to locate the corona discharge in the outdoor insulation, which has an angular error of 5.32% at 30 m distance [23]. Yu et al. propose an improved near-field acoustic holography (NAH) algorithm to visualize the measured sound field in order to locate the audible sound caused by transformer discharge faults [24]. They use a 112-channel microphone array to obtain a clear acoustic imaging map of the transformer discharge location with high resolution. There is no doubt that the acoustic localization performance is critical to the reliability of these PD localization systems and is the focus of research. Related studies are still ongoing. From our point of view, beam-forming is a promising method because of its fast computation speed and suitability for measurements over long distances and medium-high frequency signals [25,26]. However, the conventional beam-forming (CBF) algorithm has poor performance in spatial resolution and suffers serious sidelobe interferences [27]. These shortcomings obstruct its application in PD localization. Several improved algorithms are developed to overcome the limitations of CBF [28,29,30,31], and are expected to be employed for localizing the PD.

In this work, an acoustic localization sensor is proposed for partial discharge localization. This sensor uses a microelectromechanical system (MEMS) microphone array to collect the acoustic waves generated by the PD source and obtains the location of the PD through the beam-forming algorithm. MEMS microphones are particularly attractive due to their low cost, small size, and compatibility with the complementary metal-oxide-semiconductor (CMOS) fabrication process. With these advantages, they are gradually replacing other types of microphones (such as electret condenser microphones) as the first choice for smartphones, wireless earphones, and microphone arrays. In the field of PD detection, MEMS microphones can help users construct compact and reliable detection systems. The MEMS microphone array consists of eight commercial MEMS microphones (SPV08A0LR5H-1, Knowles) with an aperture size of about 0.1 m × 0.1 m. A random topology is designed to improve the acoustic performance of the array. It is compared with several common array topologies in the simulation, and the results show that it has relatively optimal performance. With respect to the localization algorithm, a deconvolution method called Fourier-based fast iterative shrinkage-thresholding algorithm (FFT-FISTA) is applied, because it can effectively improve spatial resolution and suppress sidelobes [32]. Through scanning the specified measurement area, an acoustic imaging map can eventually be obtained to confirm the presence and location of PD. The feasibility and performance of the proposed scheme are evaluated in both simulation and experiment. In the experiment section, two setups are considered. For the first setup, an ultrasonic transducer is acted as the acoustic source for direct comparison with the simulation results. In the second setup, a partial discharge test platform is built for actual PD localization test. Simulation and experiment results demonstrate that the proposed scheme has high spatial resolution and effectively eliminates sidelobe contaminations. Furthermore, the localization precision is evaluated, with an average error of 0.04 m. Overall, this work achieves satisfactory localization performance using a miniaturized, low-cost acoustic localization sensor.

## 2. The Design of Acoustic Localization Sensor

### 2.1. The Principle of Acoustic Beam-Forming

**Figure 1:**
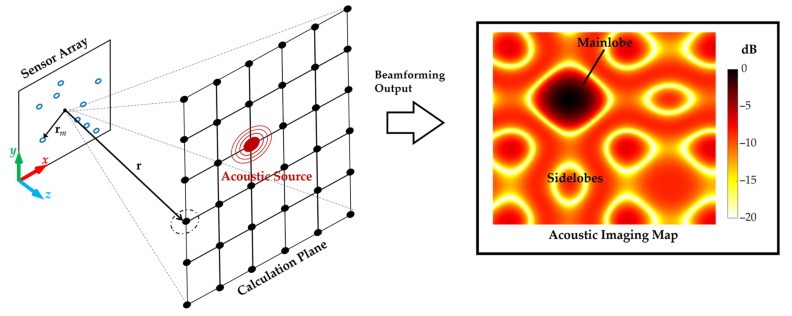
Beam-forming method and its coordinate system.

As is shown in Figure 1, an array with *M* sensors at a certain distance from the acoustic source is used for measurement, and the position of the *m*th sensor relative to the center of the array is **r***_m_*. The basic principle of beam-forming is to scan the space based on signal delay compensation and weighted summation. It starts by creating an acoustic calculation plane in the desired measurement area and discretizing the plane into a grid with *N* points distributed at certain intervals, with the point positions represented by **r**. The signals collected by each sensor are back-propagated to the point based on the corresponding delay and ultimately summed, which is a process known as “focusing”. The output of each point is plotted on an acoustic imaging map to indicate the source location. Consequently, the outputs at the corresponding position of the acoustic source are enhanced to form main lobes, and the outputs at the other positions are attenuated to form sidelobes. The cross-spectral imaging function (CSIF) is one of the conventional beam-forming (CBF) algorithms, which is widely used [33]. The output of CSIF can be written as follows:

(1) $$b\left(r\right)=\frac{1}{M}\frac{v\left(r\right)Cv^{H}\left(r\right)}{\sqrt{u\left(r\right)Eu^{H}\left(r\right)}}$$

where **E** is *M* × *M* matrix with all elements of 1, **C** is the cross-spectral matrix of the sound pressure signals. $v\left(r\right)=\left[v_{1}\left(r\right),v_{2}\left(r\right),\ldots v_{m}\left(r\right),\ldots v_{M}\left(r\right)\right]$ is the steering row vector, $v_{m}\left(r\right)=e^{-jk\left|r-r_{m}\right|}/\left|r-r_{m}\right|$, where k=2πf/c is wavenumber, *f* is frequency, *c* is velocity of the acoustic wave. u(r) can be given by $u\left(r\right)=\left[{\left|v_{1}\left(r\right)\right|}^{2},{\left|v_{2}\left(r\right)\right|}^{2},\ldots {\left|v_{m}\left(r\right)\right|}^{2},\ldots {\left|v_{M}\left(r\right)\right|}^{2}\right]$. “${\left(\cdot \right)}^{H}$” denotes conjugate transpose.

### 2.2. Sensor Array Design

The array performance has a large impact on the localization results [34], so it is necessary to focus on the design of the sensor array. The performance factors affected by the array design mainly include spatial resolution, sidelobe distributions and levels, and spatial aliasing [35]. Specifically, the spatial resolution refers to the ability to discriminate between two sources close to each other. Spatial resolution can be calculated with the Rayleigh formula and is mainly reflected by the size of the main lobe in the imaging map, and a narrower main lobe means a higher resolution. The presence of high-level sidelobes contaminates the imaging map and makes the results of the acoustic source localization uncertain. The spatial aliasing effect may produce ghost sources at other positions that are similar to the real sources, owing to the inability to satisfy the spatial sampling theorem. As a result, the upper frequency of the signal that the array can resolve will be limited. Hence, what is reflected by these factors can be used to evaluate and guide the array design as well as optimization efforts. In addition, it is also necessary to consider the constraints on the cost and size of the array in practical applications. A small aperture array with a limited number of sensors not only can be easily carried and efficiently applied to many complex environments, but it can also effectively reduce costs and system complexity.

**Figure 2:**
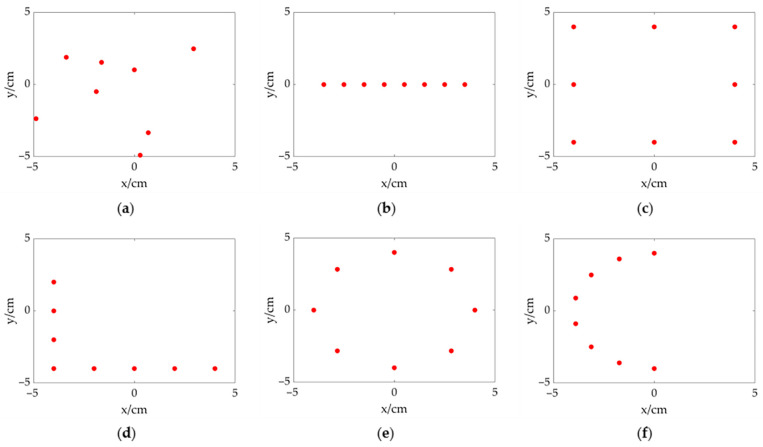
Array topologies (the red dots represent sensors). (**a**) proposed random topology; (**b**) line topology; (**c**) rectangular topology; (**d**) L-shaped topology; (**e**) circular topology; (**f**) semi-circular topology.

**Figure 3:**
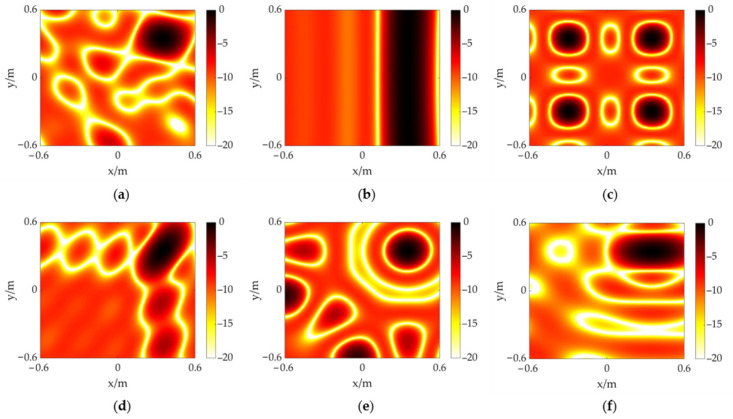
The CSIF results of different topologies at 3 m away from an acoustic source at (0.35, 0.35). (**a**) proposed random topology; (**b**) line topology; (**c**) rectangular topology; (**d**) L-shaped topology; (**e**) circular topology; (**f**) semi-circular topology.

In this section, an array model is constructed considering the above factors. The array size is set at approximately 0.1 m × 0.1 m, allowing for easy portability and placement. The number of sensors is determined to be eight for reducing the processing burden and cost. In order to enhance the acoustic performance, an array topology is determined in a random way, which is shown in Figure 2a. The results of acoustic source localization based on the CSIF are analyzed through a simulation employing a MATLAB program to evaluate the designed topology. Meanwhile, several common array topologies (including line array, rectangular array, circular array, L-shaped array, semi-circular array) are used for comparison, which are shown in Figure 2b–f. These topologies maintain a similar array size to the proposed one, and the number of array elements is also set to eight. Figure 3 shows the results of acoustic source localization for different topologies when the measuring distance is 3 m. The size of the calculation plane is 1.2 m × 1.2 m with focus points at a spacing of 0.01 m. A single acoustic source with the frequency of 40 kHz is placed at (0.35, 0.35). The output of CSIF is expressed in dB, and its maximum value is normalized. The dynamic range is set as 20 dB. The localization result of line array is shown in Figure 3a, which can only implement source identification in one dimension. Figure 3b,d show that the rectangular array and the circular array produce ghost sources in maps because of spatial aliasing. Although L-shaped and semi-circular arrays can identify a source in the 2D plane, as shown in Figure 3c,e, their main lobes are slightly larger than the random array’s. In addition, the sidelobe distribution and level are well improved through the semi-circular array and the random array. In a summary, the designed random topology meets the basic localization requirements and has the best performance compared to these common array topologies.

**Figure 4:**
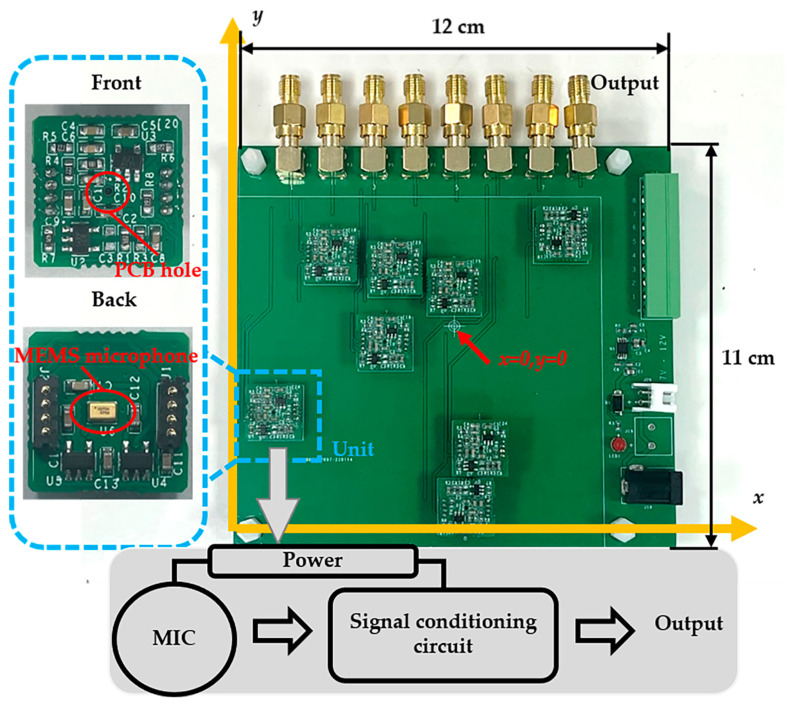
Hardware of the MEMS microphone array (with a size of 12 cm × 11 cm).

The corresponding hardware is designed according to the above array model, as shown in Figure 4. The entire hardware size is 12 cm × 11 cm. The used acoustic sensor is a MEMS microphone (SPV08A0LR5H-1, Knowles), which is a miniature, high-performance, low power, matched-sensitivity bottom-port silicon microphone. The main parameters of SPV08A0LR5H-1 are summarized in Table 1. The acoustic wave signal is converted to a voltage by the MEMS microphone, then amplified and filtered before output. Through utilizing the gap between the MEMS microphones, the signal conditioning circuit is integrated with the microphone on a small PCB unit known as an “Unit” in the subset of Figure 4. The MEMS microphone is mounted on the back of the unit, and a PCB hole is made in the middle of the unit for the acoustic port at the bottom of the MEMS microphone to be exposed. The signal conditioning circuit has a gain of about 36 dB and a frequency range of 600 Hz to 57 kHz.

### 2.3. FFT-FISTA

CBFs, such as CSIF, produce a wide main lobe at the location of the acoustic source, resulting in low spatial resolution and high-level sidelobes at positions where there is no acoustic source. Deconvolution algorithms have been developed to get around these restrictions. The output of CBF can be approximated as the convolution of the actual acoustic source distribution and the array point spread function (PSF). The aim of deconvolution is to recover the actual acoustic source distribution from the output of CBF; that is, to eliminate the influence of the PSF, which can obviously improve spatial resolution and suppress sidelobes.

Assume that there are several acoustic sources in the space and each source is incoherent. The cross-spectral of received signals is given by

(2) $$C=\sum_{r_{i}}s\left(r_{i}\right)v^{H}\left(r_{i}\right)v\left(r_{i}\right)$$

which is equivalent to the sum of the cross-spectral from each acoustic source. **r***_i_* is the location of the source. *s*(**r***_i_*) is sound pressure contribution at the center of the array.

The array PSF, which is defined as the beam-forming contribution at focus points produced by the point sound source **r***_i_* with unit intensity, can be given by

(3) $$\varphi_{psf}\left(r|r_{i}\right)=\frac{1}{M}\frac{{\left|r_{i}\right|}^{2}v\left(r\right)v^{H}\left(r_{i}\right)v\left(r_{i}\right)v^{H}\left(r\right)}{\sqrt{u\left(r\right)Eu^{H}\left(r\right)}}$$

Depending on Equations (2) and (3), Equation (1) can be modified as

(4) $$b\left(r\right)=\sum_{r_{i}}s\left(r_{i}\right)\varphi_{psf}\left(r|r_{i}\right)$$

Through assuming that the PSF is shift-invariant, the fast Fourier transform (FFT) can be used to accelerate the computation and thus improve efficiency. The shift-invariant PSF only depends on the relative location of the observation point and the source. Then,

(5) $$b\left(r\right)=\sum_{r_{i}}s\left(r_{i}\right)\varphi_{psf}\left(r-r_{i}\right)$$

The convolution operation can be converted to a wavenumber domain product with FFT, which can be written as

(6) $$B=S*P=F^{-1}\left(F\left(S\right)\circ F\left(P\right)\right)$$

where **B** and **S** are matrices consisting of CBF output and sound pressure contribution at each focus point, respectively. $P=\left[\varphi_{psf}\left(r-r_{i}\right)\right]$ is matrix with shift-invariant PSF. Moreover “ ∗ ” is convolution symbol and “ ∘ ” is Hadamard product. *F* and $F^{-1}$ represent 2D Fourier transform and its inverse. The sound pressure contribution **S** is calculated by minimizing the following expression

(7) $$\frac{1}{2}{\left\|F^{-1}\left(F\left(S\right)\circ F\left(P\right)\right)-B\right\|}_{Fro}^{2}$$

where ${\left\|\cdot \right\|}_{Fro}$ is the Frobenius norm, each element of the matrix **S** is greater than or equal to zero. The process of the FFT-FISTA is summarized in Algorithm 1.
**Algorithm 1** FFT-FISTA**Input:** shift-invariant PSF matrix **P**, the output matrix of CSIF **B**, the number of iterations *K*;

1: **Initialization:**
$S^{\left(0\right)}=0$, $Y^{\left(1\right)}=S^{\left(0\right)}$, $\tau^{\left(1\right)}=1;$

2: **loop**

3:  $S^{\left(t\right)}=\rho_{+}\left(Y^{\left(t\right)}-F^{-1}\left(F\left(P_{R}\right)\circ F\left(F^{-1}\left(F\left(Y^{\left(t\right)}\right)\circ F\left(P\right)\right)-B\right)\right)/L\right);$

4:  $\tau^{\left(t+1\right)}=\frac{1}{2}\left(1+\sqrt{1+4{\left(\tau^{\left(t\right)}\right)}^{2}}\right);$

5:  
$Y^{\left(t+1\right)}=S^{\left(t\right)}+\left(\left(\tau^{\left(t\right)}-1\right)/\tau^{\left(t+1\right)}\right)\left(S^{\left(t\right)}-S^{\left(t-1\right)}\right);$

6:  **if **
t>K
**then**

7:   **break**;

8:  **end if**

9:  t=t+1;

10: **end loop**

**Output:**
$S^{\left(t\right)}$;

In Algorithm 1, *ρ*_+_ denotes the Euclidean projection of **S** onto the non-negative orthant, **P**_*R*_ is obtained by a 180° rotation of **P**. *L* is a Lipschitz constant [32].

It is worth noting that, since the deconvolution algorithm accelerated by the fast Fourier transform is based on the assumption that the point spread function is shift-invariant, the PSF at the center of the computational plane is typically utilized as the shift-invariant PSF. However, the shift-invariant PSF is a good approximation only when it is close to the center of the computational plane. As the acoustic source deviates from the center of the plane, the imaging results will deteriorate. To improve this problem, an irregular focus point grid proposed in [37] is used instead of the traditional regular one.

## 3. Simulation of the Acoustic Localization Sensor

**Figure 5:**
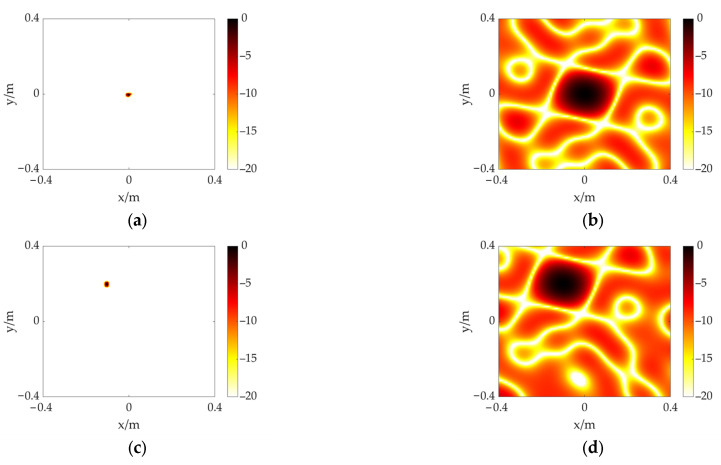
The simulation results of a single acoustic source at different positions including (0, 0) and (−0.10, 0.20). Distance is 2 m. (**a**) FFT-FISTA, (0, 0); (**b**) CSIF, (0, 0); (**c**) FFT-FISTA, (−0.10, 0.20); (**d**) CSIF, (−0.10, 0.20).

In this section, the simulation is performed through MATLAB to evaluate the proposed scheme. The corresponding array model in Section 2 and the coordinate system in Figure 1 are established. A 40 kHz acoustic source with a known location is set in the calculation plane (*x*-*y* plane). Two measurement distances (z=2,3 m, respectively) are considered in this simulation. In the case of a measurement distance of 2 m, the acoustic imaging maps of the proposed scheme are shown in Figure 5a,c, where the acoustic source is set at (0, 0) and (−0.10, 0.20), respectively. The size of the calculation plane is 0.8 m × 0.8 m with the irregular focus point grid mentioned in the above section. The number of deconvolution iterations is 1500, and the dynamic range is 20 dB. In addition, the results based on the CSIF algorithm are used to compare and analyze, as shown in Figure 5b,d. It can be found that both methods are able to identify the target acoustic source. However, the main lobe of the CSIF-based scheme covers large areas, and the sidelobe interferences are serious, leading to contamination of the imaging map, while the FFT-FISTA-based scheme significantly reduces the size of the main lobe, improves the spatial resolution, and effectively suppresses the sidelobes, which provides better performance.

**Figure 6:**
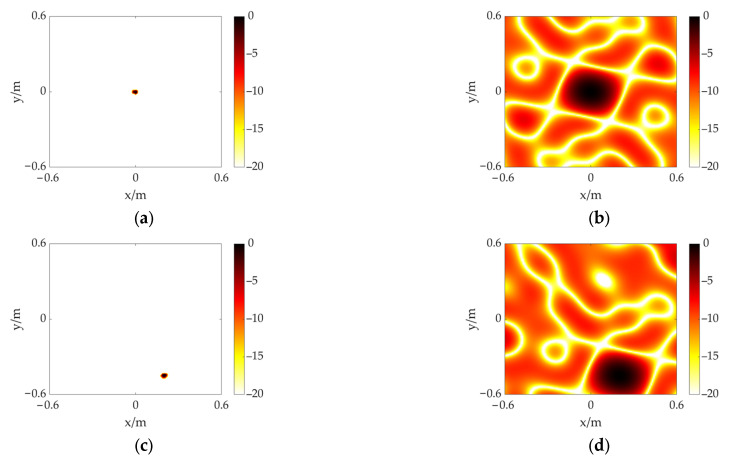
The simulation results of a single acoustic source at different positions including (0, 0) and (0.20, −0.45). Distance is 3 m. (**a**) FFT-FISTA, (0, 0); (**b**) CSIF, (0, 0); (**c**) FFT-FISTA, (0.20, −0.45); (**d**) CSIF, (0.20, −0.45).

Then, the measuring distance is increased to 3 m, and the acoustic imaging maps of the FFT-FISTA-based scheme and the CSIF-based scheme are shown in Figure 6a,c and Figure 6b,d, where the acoustic source is set at (0, 0) and (0.20, −0.45), respectively. The size of the calculation plane is adjusted to 1.2 m × 1.2 m as the distance increases, and other settings remain unchanged. From Figure 6, the same conclusion as the previous case can be drawn. According to the simulation results, the proposed scheme not only identifies and locates the target source, but it also achieves a high resolution with effective sidelobe interference elimination in the measurement range.

## 4. Experiment and Discussion

In the following section, two experimental setups are built to validate the feasibility and performance of the acoustic localization sensor. The measurement system is composed of the designed MEMS microphone array, a data acquisition equipment (SPIDER-80X, Crystal Instruments, Santa Clara, CA, USA) with 8 channels for simultaneous acquisition, and a personal computer to process acquired signals. The sampling frequency is set to 102,400 Hz. The conditioned signals from the microphone array are digitized by the SPIDER-80X and then loaded into the PC. The localization processing is carried out through algorithm programs in MATLAB.

**Figure 7:**
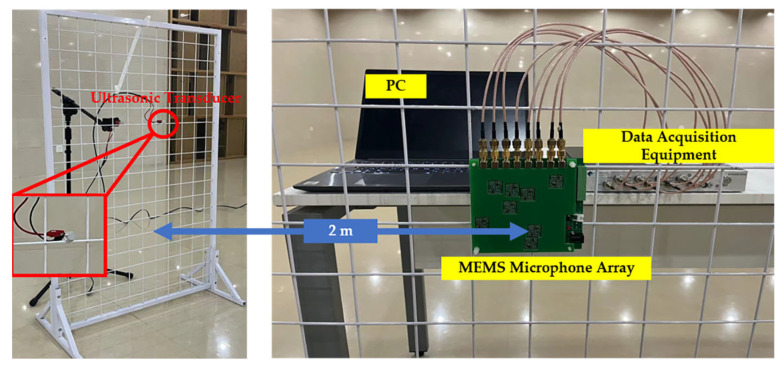
The setup of acoustic source localization experiment. The acoustic wave is produced by an ultrasonic transducer shown in the left picture. The data acquisition system is shown on the right side of the figure.

**Figure 8:**
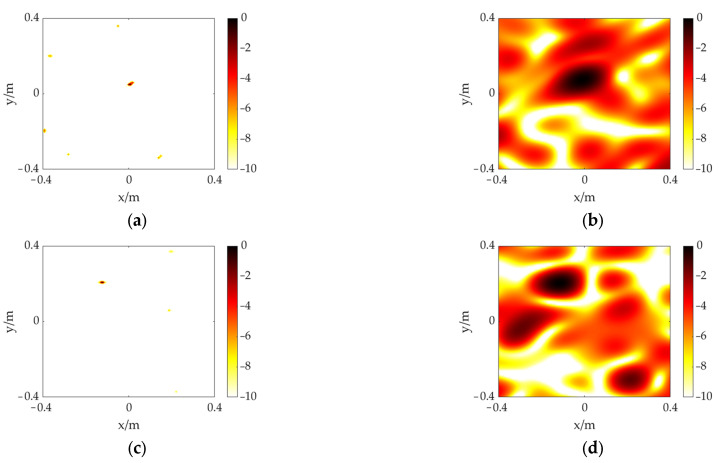
The experiment results of an acoustic source produced by an ultrasonic transducer at different positions. (**a**) FFT-FISTA, (0, 0); (**b**) CSIF, (0, 0); (**c**) FFT-FISTA, (−0.10, 0.20); (**d**) CSIF, (−0.10, 0.20).

In the first experimental setup, for a direct comparison with simulations, a signal generator is used to provide electrical excitation for the ultrasonic transducer, whose center frequency is 40 kHz, to radiate the acoustic wave outward as an acoustic source. A 40 kHz sine wave is applied to the transducer. The array is placed at a distance of 2 m from the transducer to sample the acoustic field. The first setup is shown in Figure 7. The size of the calculation plane is set to 0.8 m × 0.8 m with the irregular focus point grid, and the transducer is set at (0, 0) and (−0.10, 0.20), respectively. The iterations of the deconvolution algorithm are 1500, and the dynamic range is set at 10 dB. The FFT-FISTA-based scheme can correctly identify the target acoustic source from the ultrasonic transducer, as shown in Figure 8a,c. Moreover, it shows a sufficiently small main lobe with a high spatial resolution, while the sidelobes are almost completely suppressed. Nevertheless, as shown in Figure 8b,d, the main lobe coverage area of the CSIF-based scheme is significantly increased, resulting in a poor spatial resolution. The contamination of imaging maps caused by sidelobes is severe. The results of this experiment are consistent with the simulation.

**Figure 9:**
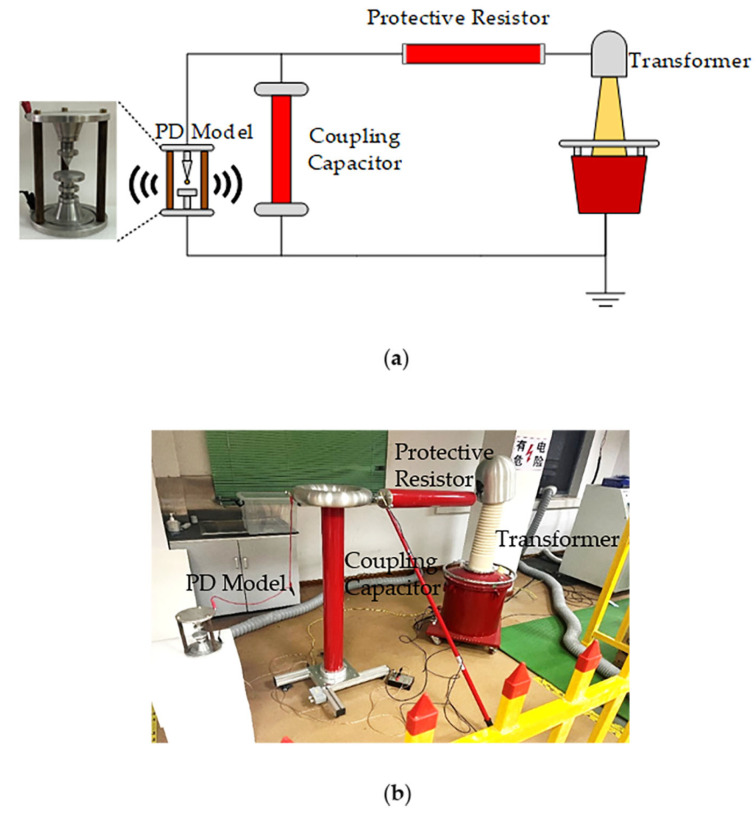
(**a**) The schematic diagram of PD test platform. (**b**) PD experiment site.

In the second experimental setup, a PD test platform is built for PD localization testing. As can be seen in Figure 9, the experimental platform mainly consists of a 10 kVA/150 kV transformer, a test console, a 150 kV/500 PF coupling capacitor, a 5.3 kΩ resistor for over-current protection, and a cone-plate electrode model. The cone-plate electrode model is used to generate a partial discharge with a 50 Hz AC voltage applied to the high-voltage electrode of the discharge model.

**Figure 10:**
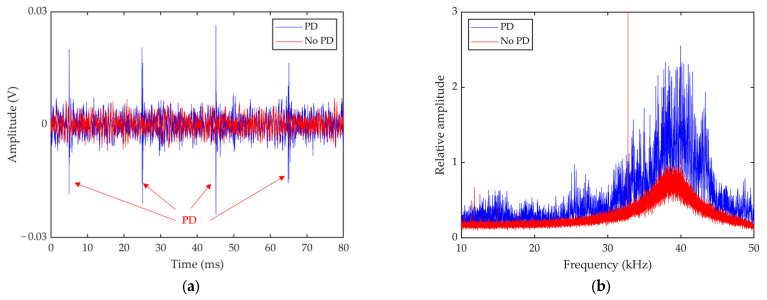
Time domain signal (**a**) from a microphone channel and the corresponding frequency domain information (**b**).

**Figure 11:**
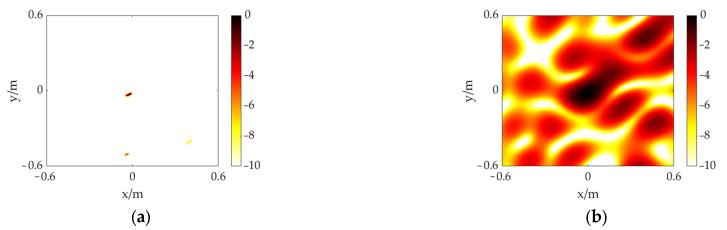

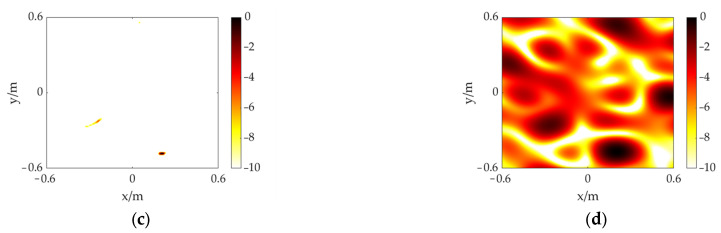
The experiment results of a PD source at different positions. (**a**) FFT-FISTA, (0, 0); (**b**) CSIF, (0, 0); (**c**) FFT-FISTA, (0.20, −0.45); (**d**) CSIF, (0.20, −0.45).

The distance between two electrodes is 10 mm. The voltage is raised through the high-voltage test console to produce a significant discharge phenomenon. Considering the experimental safety distance and the space limitation of the laboratory, the measurement distance is set to 3 m. After connecting the experimental equipment and increasing the voltage, an obvious discharge phenomenon can be observed. The acquired signal from one of the channels is shown in Figure 10. The time and frequency domains reveal distinct discharge features, proving the PD can be effectively detected by our sensor. The size of the calculation plane is set to 1.2 m × 1.2 m. The PD model is located at (0, 0) and (0.20, −0.45), respectively. The other settings remained the same as in the previous experiment. As shown in Figure 11b,d, the CSIF-based scheme performs poorly, and high-level sidelobes appear in the maps, making identification and location of the PD source impossible. In contrast, the PD source can be clearly identified from Figure 11a,c. Moreover, due to the presence of environmental noise, slight sidelobes appear in the experimental results relative to the simulation. However, these faint sidelobes have almost no impact on localization results. Therefore, the FFT-FISTA-based scheme still demonstrates satisfactory performance.

On these bases, the localization accuracy of this acoustic localization senor is analyzed and summarized in Table 2. The absolute error is obtained from the distance between the locating position (0 dB in the imaging map) and the actual position of the source. According to Table 2, these errors are acceptable for the corresponding detection distances.

## 5. Conclusions

In this paper, an acoustic localization sensor is designed for partial discharge localization, involving the design of a sensor array and the application of the beam-forming algorithm. Eight MEMS microphones are used in a random topology to form a small aperture array, which makes the hardware miniaturized and cost-effective. The designed random topology has the best performance compared to some common array topologies, providing a basis for reliable acoustic localization. The acoustic source is located by FFT-FISTA, which is based on the principle of deconvolution to eliminate the effect of the array point spread function in the conventional beam-forming algorithm. Simulations and experiments with a sine wave source indicate that our scheme significantly improves the spatial resolution and suppresses the sidelobe interferences. Finally, the feasibility and satisfactory performance of the presented scheme are validated on the PD test platform. The average error of localization is about 0.04 m. The work in this paper can be combined with an optical camera to locate the PD intuitively and conveniently. There are some issues that need to be further studied. First, there are several types of PD (only one type is considered in this paper), and different PD types exhibit distinct signal features. We will further investigate these types and better adapt the present effort to the localization of these various PD types. In addition, PD type identification is also an important task in insulation condition monitoring that deserves in-depth research. Secondly, measurements in various equipment and field environments need to be taken into account, and our scheme needs to be adjusted and optimized according to the actual situation. Nevertheless, our ultimate aim is to make the PD acoustic localization more reliable. Moreover, in future work, we intend to integrate the embedded computing platform with the sensor array for a more efficient and compact system.

## Figures and Tables

**Table 1:** The main parameters of SPV08A0LR5H-1 [36].

Parameter	Specification
Sensitivity	−42 dBV/Pa
Operating frequency range	100 Hz~80 kHz
Center frequency	40 kHz
Package dimensions	2.75 mm × 1.85 mm × 0.90 mm

**Table 2:** The localization results and errors.

Acoustic Source	Measurement Distance (m)	Actual Position (m)	Locating ^1^ Position (m)	Absolute Error (m)
transducer	2	(0, 0)	(0, 0.05)	0.05
transducer	2	(−0.10, 0.20)	(−0.12, 0.21)	0.02
PD model	3	(0, 0)	(0.03, 0.03)	0.04
PD model	3	(0.20, −0.45)	(0.21, −0.49)	0.04

^1^ The locating position refers to the location of the maximum value (0 dB in the imaging map).

## Data Availability

Not applicable.
